# Virus-derived transgenes expressing hairpin RNA give immunity to *Tobacco mosaic virus *and *Cucumber mosaic virus*

**DOI:** 10.1186/1743-422X-8-41

**Published:** 2011-01-27

**Authors:** Qiong Hu, Yanbing Niu, Kai Zhang, Yong Liu, Xueping Zhou

**Affiliations:** 1 State Key Laboratory of Rice Biology, Institute of Biotechnology, Zhejiang University, Hangzhou, 310029, P.R. China; 2 Hangzhou Wanxiang polytechnic, Hangzhou, 310023, P.R. China

## Abstract

**Background:**

An effective method for obtaining resistant transgenic plants is to induce RNA silencing by expressing virus-derived dsRNA in plants and this method has been successfully implemented for the generation of different plant lines resistant to many plant viruses.

**Results:**

Inverted repeats of the partial *Tobacco mosaic virus *(TMV) movement protein (MP) gene and the partial *Cucumber mosaic virus *(CMV) replication protein (Rep) gene were introduced into the plant expression vector and the recombinant plasmids were transformed into *Agrobacterium tumefaciens*. *Agrobacterium*-mediated transformation was carried out and three transgenic tobacco lines (MP16-17-3, MP16-17-29 and MP16-17-58) immune to TMV infection and three transgenic tobacco lines (Rep15-1-1, Rep15-1-7 and Rep15-1-32) immune to CMV infection were obtained. Virus inoculation assays showed that the resistance of these transgenic plants could inherit and keep stable in T_4 _progeny. The low temperature (15℃) did not influence the resistance of transgenic plants. There was no significant correlation between the resistance and the copy number of the transgene. CMV infection could not break the resistance to TMV in the transgenic tobacco plants expressing TMV hairpin MP RNA.

**Conclusions:**

We have demonstrated that transgenic tobacco plants expressed partial TMV movement gene and partial CMV replicase gene in the form of an intermolecular intron-hairpin RNA exhibited complete resistance to TMV or CMV infection.

## Background

The plant disease caused by *Tobacco mosaic virus *(TMV) or *Cucumber mosaic virus *(CMV) is found worldwide. The two viruses are known to infect more than 150 species of herbaceous, dicotyledonous plants including many vegetables, flowers, and weeds. TMV and CMV cause serious losses on several crops including tobacco, tomato, cucumber, pepper and many ornamentals. During the last decade, several laboratories have tried to introduce resistance to TMV or CMV by genetic engineering. Virus resistance in plants containing virus-derived transgene, usually by the expression of functional or dysfunctional coat protein, movement protein or polymerase gene, has been widely reported. The TMV coat protein gene was used in the first demonstration of virus-derived, protein-mediated resistance in transgenic plants [1]. Pathogen-derived resistance for CMV often showed only partial resistance or very narrow spectrum of resistance to the virus [2].

RNA silencing or post-transcriptional gene silencing (PTGS), developed during plant evolution, functions as a defense mechanism against foreign nucleic acid invasions (viruses, transponsons, transgenes) [3]. Since the phenomenon of RNA silencing was first observed by Napoli [4], research has been carried out to elucidate its mechanism. PTGS is a mechanism closely related to RNA interference, which is involved in plant defense against virus infection [5,6]. It was found that when a inverted repeated sequences of partial cDNA from a plant virus are introduced into host plants for expression of dsRNA and induction of RNA silencing, the transgenic plants can silence virus corresponding gene and are resistant to virus infection [7,8]. More than 90% of transgenic *Nicotiana benthamiana *lines were resistant to the virus when engineered with hairpin constructs using *Plum pox virus P1 *and *Hc-Pro *genes sequences under the *35S*-cauliflower mosaic virus promoter [9]. For the current study, we expressed the partial TMV movement protein (MP) gene and the partial CMV replication protein (Rep) gene in the form of an intermolecular intron-hairpin RNA in transgenic tobacco. We analyzed the resistance of T_0 _to T_4 _transgenic plants. We found that the two T_4 _transgenic lines with single copy were completely resistant to the corresponding virus, and the viral resistance of transgenic plants did not be affected by the low temperature (15℃).

## Results

### Transformation and analysis of T_0 _plants

**Figure 1:**
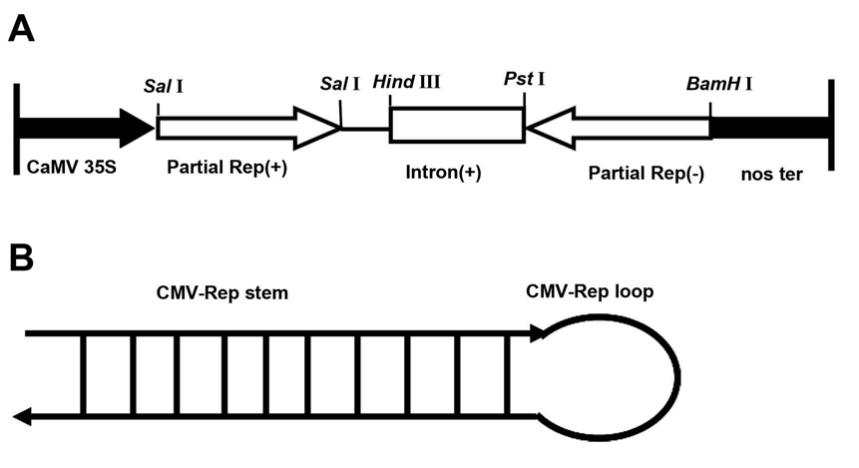
**(A) Schematic map of the T-DNA region of pBIN-CMVΔ*Rep*(i/r) and (B) Diagram of self-complementary (hairpin) RNA produced by pBIN-CMVΔ*Rep*(i/r)**. CaMV 35S: Cauliflower mosaic virus 35S promoter; nos ter: nopaline synthase terminator.

Transgenic tobacco plants expressing hairpin RNA derived from TMV Δ*MP *or CMV Δ*Rep *gene were generated by *Agrobacterium tumefaciens*-mediated transformation (Figure 1). Thirty T_0 _transgenic plant lines containing TMV *MP *sequences and twenty T_0 _transgenic plant lines containing CMV *Rep *sequences were obtained by kanamycin selection. The specific DNA fragment was amplified in all transgenic lines by PCR using primers TMV MP-F1 and TMV MP-R1 specific for TMV *MP *or primers Δ*Rep*-F and Δ*Rep*-R specific for CMV *Rep *gene (data not shown). Southern blot analyses of selected transgenic lines indicated that the *MP *or *Rep *gene fragment was integrated into the genomic DNA and the copy number of the foreign gene was estimated to be one to more than five (Table 1).

**Table 1:** Testing of T_0 _and T_1 _transgenic plants for TMV or CMV resistance.

T_0 _line number	T_0 _reaction to TMV^a^	T_0 _reaction to CMV	T_0 _copy number of transgene (Southern)	T_1 _segregation immune:susceptible
MP16	Immune		1	36:14*
MP31	Immune		>3	31:19
MP39	Immune		2	39:11
MP53	Immune		2	40:10
MP36	Susceptible		1	0:50

Rep15		Immune	1	38:12*
Rep17		Immune	2	30:20
Rep25		Resistant	>5	24:26
Rep53		Resistant	>3	29:21
Rep727		Susceptible	3 ~ 4	0:50

^a^Immune indicated no detectable symptom and no virus particles were detected. Resistant indicated mild symptom and virus was detected. Susceptible indicated clear mosaic symptoms in the entire leaves.

* The segregation for TMV or CMV resistance vs. susceptibility conforms to a 3: 1 ratio for a single dominant locus (χ2 test, P > 0.05).

### Resistant response of T_0 _to T_4 _transgenic progenies to infection of TMV or CMV

The successive generation seeds were obtained by self-pollination from inoculated plants and the progenies of different lines were gained simultaneously for further analyses. Seedlings per each line were randomly taken from the resultant regenerates for virus inoculation tests. The T_1 _progenies of T_0 _parental lines, MP16, MP31, MP39, MP53, Rep15, Rep17, Rep25 and Rep53 contained some plants that were immune and others that were susceptible, whereas the T_0 _parental line MP36 or Rep727 which was susceptible to the virus yielded only susceptible progenies in successive generations (Table 1). The progeny of T_0 _lines MP16 and Rep15 was confirmed to a have a segregation ratio of 3:1 (immune: susceptible), suggesting the presence of a single dominant transgene locus in each line, and Southern blot analysis revealed that the loci each appear to have a single transgene (Table 1).

**Figure 2:**
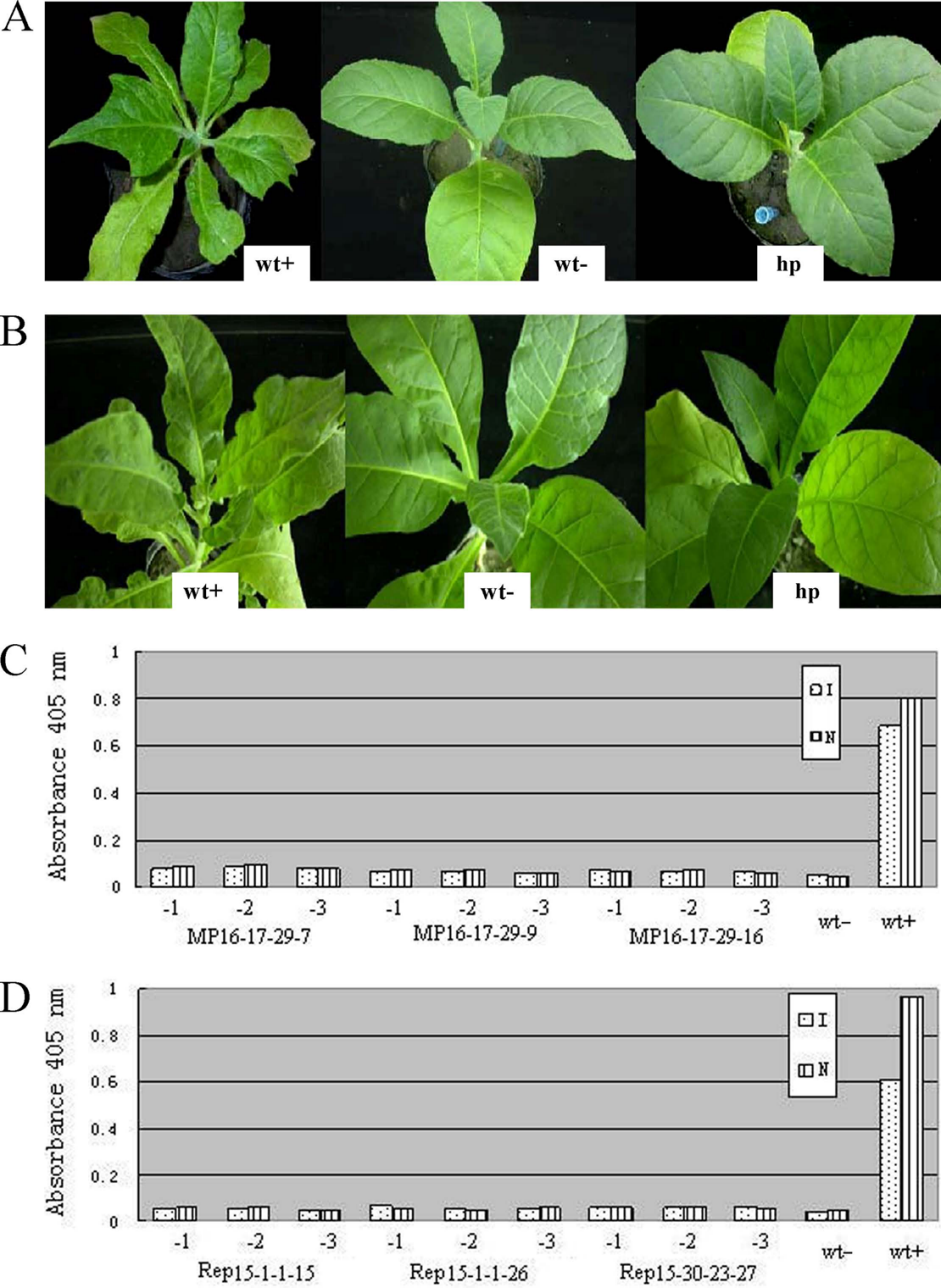
**(A, B) Reaction of T**_**4 **_**transgenic plants (hp) to TMV (A) or CMV (B) infection at three-month after virus inoculation**. Wild-type *Nicotiana tabacum *(cv. Yunyan 87) plants inoculated with buffer (wt-) or with TMV or CMV (wt+) were used as controls. (C, D) Accumulation levels of TMV (C) or CMV (D) in T_4 _transgenic plants. The 5-6 leaves stage T_4 _transgenic plants were mechanically inoculated with TMV or CMV and new emergent leaves were collected at 25 days after inoculation for ELISA. The absorbance value represents the mean value obtained from three independent ELISA assays. Plants were considered as virus infected when the corresponding absorbance values measured at 405 nm were more than two times as compared to mean absorbance values from the healthy plants. I, inoculated leaves; N, new growth leaves. wt-, wild type plant inoculated with buffer; wt+, wild type plant inoculated with TMV or CMV.

Responses to TMV or CMV infection were further examined for the phenotype of T_2_, T_3 _and T_4 _generation. Resistant T_1 _lines were randomly selected from each of the six T_0 _lines (MP16, MP31, MP39, MP53, Rep15 and Rep17) that generated both resistant and susceptible progenies and the two T_0 _lines (MP36 or Rep727) that only generated susceptible progenies were also selected. In the screening of the T_2 _generation, plants were randomly selected and inoculated with TMV or CMV. Most of the T_2 _generation plants derived from resistant T_1 _lines segregated for both resistant and susceptible phenotype, whereas all T_2 _progenies from the resistant T_1 _lines, MP16-17 and Rep15-1, were immune, which showed no any symptoms and no virus replication when measured by TAS-ELISA at 25 days after inoculation (Table 2). The resistant T_2 _lines MP16-17-3, MP16-17-29, MP16-17-58, Rep15-1-1, Rep15-1-7 and Rep15-1-32 generated only immune phenotypes in the successive T_3 _and T_4 _generations, confirming the stable inheritance of resistance (Table 2), although most of the other resistant parental T_2 _or T_3 _segregated for a few susceptible plants in T_3 _or T_4 _generations. On the contrary, all of the T_2 _progenies from susceptible T_1 _lines (MP36-17 or Rep727-1), were susceptible to TMV or CMV and did not segregate for resistance in the successive generations (Table 2). T_4 _transgenic plants kept immunity phenotypes were shown in Figure 2. The immunity transgenic plants (hp) were completely asymptomatic (Figure 2A and 2B). When samples from inoculated leaves and new emergent leaves of different immune T_4 _lines were detected with TAS-ELISA at 25 days after CMV or TMV inoculation, the absorbance value from either inoculated or new (systemic) leaves of inoculated plants were as low as negative samples (wt-) (Figure 2C and 2D), which indicated that the virus replication was prevented at local and systemic infection in transgenic immunity plants. Severe mosaic symptoms were found at 30 days after TMV or CMV inoculation on untransformed wild-type plants (wt+) (Figure 2A and 2B). The results suggest that the resistance induced by the hairpin RNA is stably inherited through self-pollination for the fourth generations.

**Table 2:** Segregation of TMV or CMV resistance over the T_2_, T_3 _and T_4 _generations of *Nicotiana tabacum *transformed with inverted repeats of the partial TMV movement protein (*MP*) gene or the partial CMV Replication protein (*Rep*) gene.

T_1 _**line**	**No. of T**_**2 **_**plants showing R/S**^**a**^		**T**_**2 **_**line**		**No. of T**_**3 **_**plants showing R/S**		**Responses of T**_**4 **_**progenies**
					
	R	S			R	S	
MP16-17	45	5	MP16-17	-3	50	0	All immune
				-29	50	0	All immune
				-58	50	0	All immune
MP31-28	36	14	MP31-28	-31	41	9	Variable (47/3)^c^
				-43	37	13	--^**d**^
				-55	40	10	--
MP39-36	39	11	MP39-36	-25	33	17	Variable (42/8)
				-37	39	11	--
				-49	38	12	--
MP53-52	43	7	MP53-52	-7	42	8	Variable (46/4)
				-21	45	5	Variable (48/2)
				-46	44	6	Variable (48/2)
MP36-17	0	50	MP36-17	-2	0	50	All susceptible
Wt+^b^	0	50	Wt+		0	50	All susceptible

Rep15-1	50	0	Rep15-1	-1	50	0	All immune
				-7	50	0	All immune
				-32	50	0	All immune
Rep15-30	46	4	Rep15-30	-7	39	11	Variable (42/8)
				-23	46	4	Variable (45/5)
				-38	42	8	Variable (48/2)
Rep15-66	36	14	Rep15-66	-7	32	18	Variable (40/10)
				-36	37	13	Variable (41/9)
				-48	35	5	Variable (38/12)
Rep17-8	21	29	Rep17-8	-15	22	28	--
				-24	0	50	All susceptible
Rep727-1	0	50	Rep727-1	-20	0	50	All susceptible
Wt+^b^	0	50	Wt+		0	50	All susceptible

^a^R indicated immune and S indicated susceptible with clear mosaic symptoms.

^b^Wt+ represented wild type *Nicotiana tabacum *inoculated with TMV or CMV.

^c^Immune/susceptible.

^d^--Not tested.

### Comparative analysis of the T_2 _or T_4 _transgene and the mode of expression in terms of resistance

**Figure 3:**
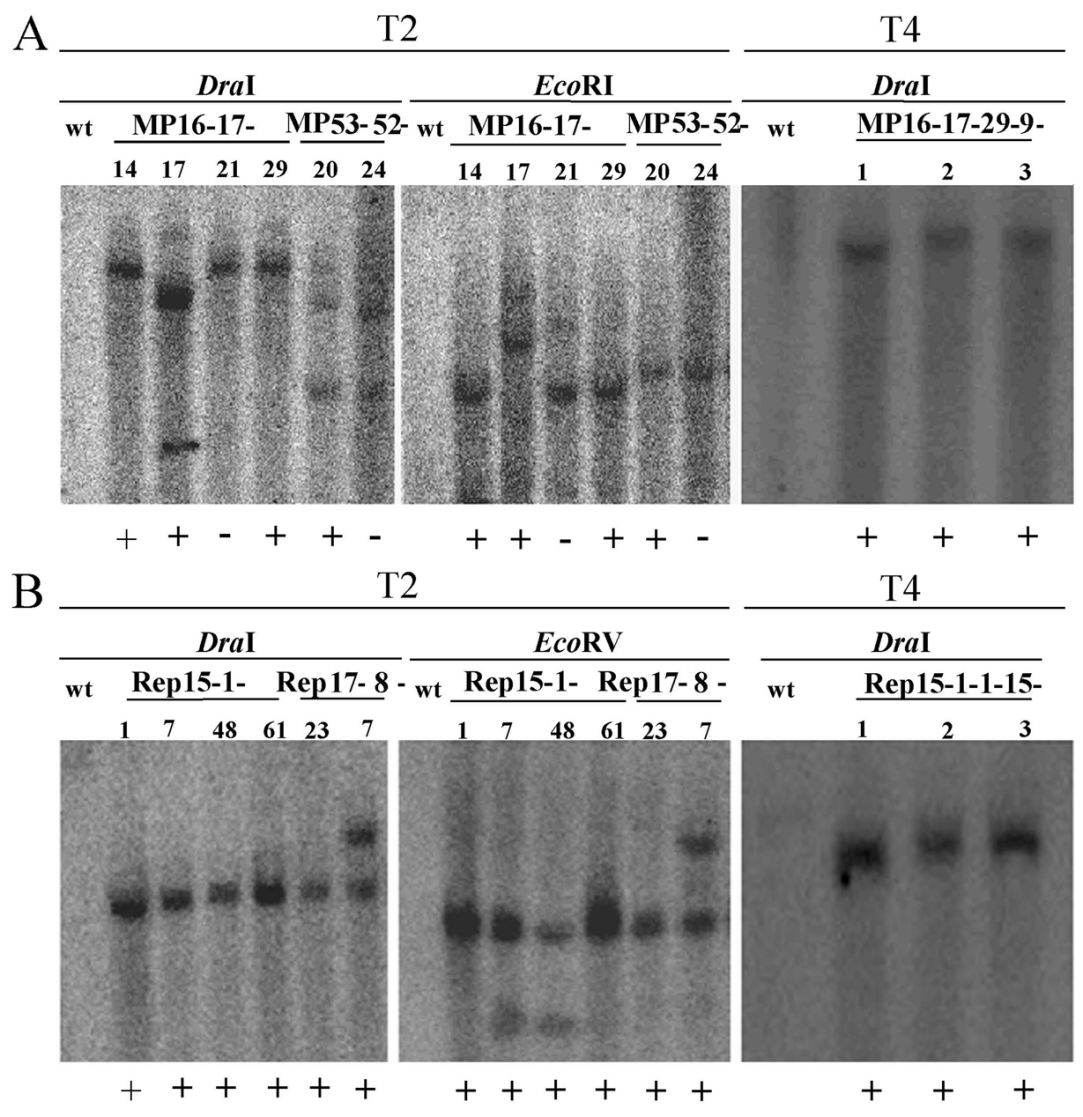
**Southern blot analyses of T**_**2 **_**and T**_**4 **_**transgenic plants expressing hairpin RNA of TMV partial *MP *(A) or CMV partial *Rep *(B)**. Genomic DNA from immune (+), susceptible (-) or wild type tobacco (wt) plants was digested with *Dra*I, *Eco*RI or *Eco*RV, and hybridized with a radioactively labeled TMV*ΔMP *(A) or CMV*ΔRep *(B) probe.

Correlation between the number of transgene insertions and the type of RNA silencing in tobacco were investigated in this study. Genomic DNA of each line was digested with *Dra*I, *Eco*RI or *Eco*RV (in the genomic DNA outside of the hairpin cDNA). The resistant T_1 _plants derived from resistant T_0 _lines (MP16, MP53 or Rep15, Rep17) carried one to two copies of transgenes by Southern blot analyses (data not shown). Then the transgene copy number of the T_2 _progenies from resistant T_1 _lines (MP16-17, MP53-52 or Rep 15-1, Rep 17-8) were also detected by Southern blot. The transgene copy number of hybridized DNA restriction fragments varied among the progenies regardless of the infection type. For example, there were immune lines containing one (Figure 3A, MP16-17-29) or two copies of transgene (Figure 3B, Rep17-8-7), but susceptible lines with one (Figure 3A, MP16-17-21) or more than three copies of transgene (Figure 3A, MP53-52-24) were also observed. So no any co-relationships between the transgene copy number and viral resistance level were found. Southern blot analysis results of T_4 _plants derived from T_3 _lines (MP16-17-29-9 or Rep15-1-1-15) which contained single copy showed that all T_4 _plants carried single copy (Figure 3).

**Figure 4:**
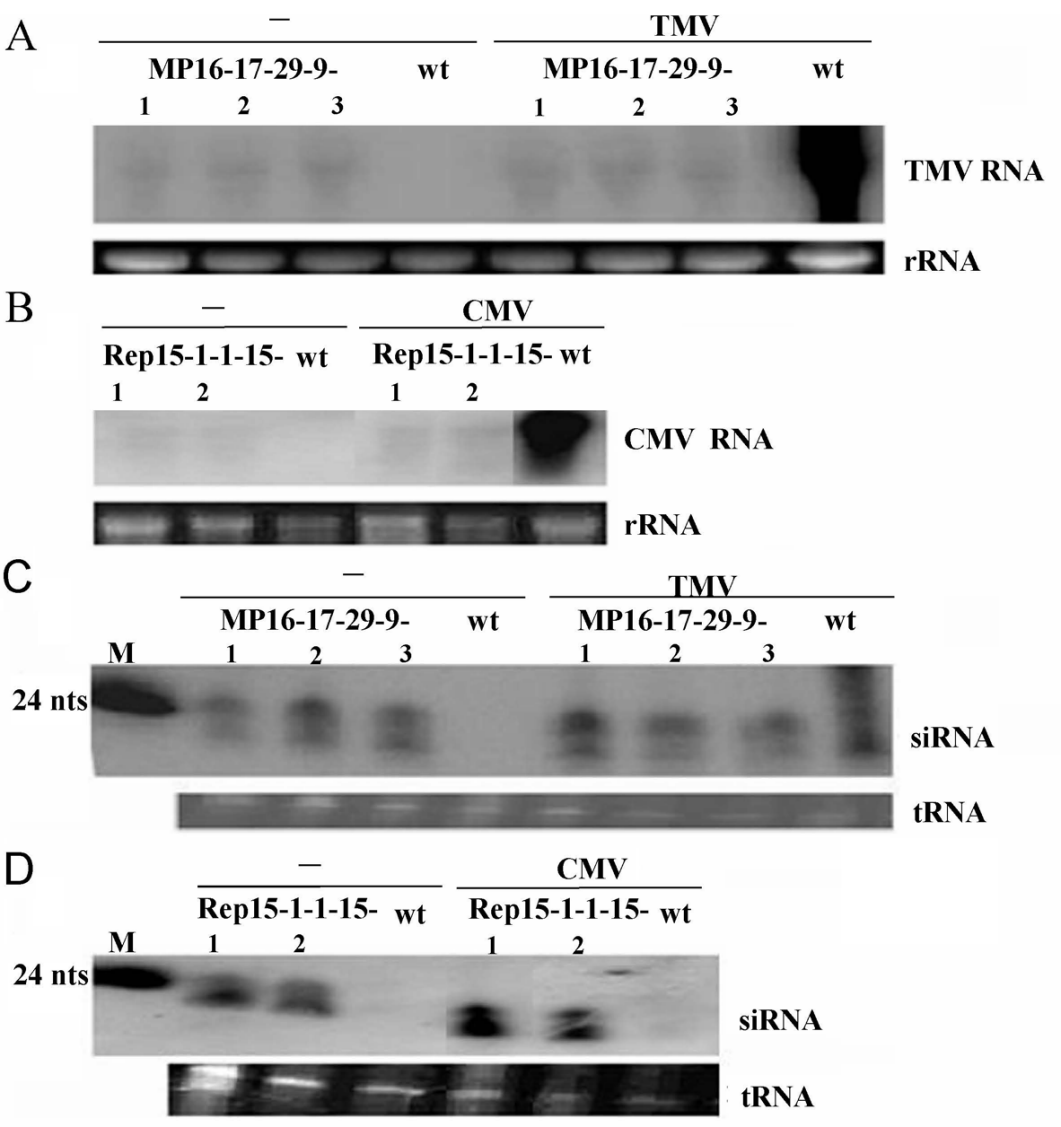
**Northern blot analyses of TMV RNA (A), CMV RNA (B), TMV siRNA (C), or CMV siRNA (D) of T**_**4 **_**transgenic plants before inoculation (-) or after inoculation with TMV or CMV**. Wild type plant (wt) was used as a control. The size of the marker DNA oligomers (24nts) was presented on the left. The lower panel shows the loading level of each RNA sample by ethidium bromide staining.

Next, we determined the accumulation of transgene-derived RNA transcripts. Northern hybridization analyses confirmed that only very little transcript of the transgene could be detected at day 25 after the virus inoculation or before virus inoculation, whereas in the wild-type infected plants, the accumulation level of the viral genomic RNA was very high (Figure 4A and 4B).

The virus-specific siRNA was detected by Northern blot analysis of low weight RNAs prepared from the leaves of T_4 _transgenic and non-transgenic tobacco plants using [α-^32^P]dCTP-labelled partial *MP *or *Rep *gene as a probe and the result showed distinct hybridization signal bands of expected size for siRNA (approximately 21-24 nts, homologous to the *MP *or *Rep *transcripts) only existed in immune transgenic plants whether virus was inoculated or not. No siRNA could be detected in healthy wild-type control plants (Figure 4C and 4D).

In our study, all the progenies from MP16-17-29-7, MP16-17-29-7 lines or Rep15-1-1-15, Rep15-1-1-26 lines did not show any symptoms of local or systemic infection during their entire life cycle, and grew normally, developed flowers, and later set fruits with normal seeds. Inoculated non-transgenic control plants showed a significant delay in flowering, stunting and less or no seeds when compared to the un-inoculated control plants. There were no differences in the plant height and seed weight between the inoculated transgenic immune plants and healthy non-transgenic plants (Table 3).

**Table 3:** T_4 _transgenic plant height and seed weight comparing with wild plant

T_3 _line	N ^a^	Reaction to virus	Height per plant (m)			Seed weight per plant (g)		
			
			Min^b^	Max^c^	Mean ± SE^f^	Min	Max	Mean ± SE^f^
MP16-17-29-7	15	immune	1.074	1.479	1.298 ± 0.101 a	1.989	3.574	3.251 ± 0.392 a
MP16-17-29-16	15	immune	1.006	1.348	1.237 ± 0.094 a	1.579	3.776	3.067 ± 0.586 a
wt+ ^d^	15	infected	0.357	0.774	0.573 ± 0.101 b	0.611	1.062	0.665 ± 0.108 b
wt ^e^	15		1.092	1.378	1.251 ± 0.074 a	2.056	3.849	3.472 ± 0.454 a

Rep15-1-1-15	15	immune	0.875	1.197	1.076 ± 0.083 a	1.774	3.207	2.879 ± 0.363 a
Rep15-1-1-26	15	immune	0.997	1.246	1.195 ± 0.065 a	1.855	3.169	2.794 ± 0.331 a
wt+	15	infected	0.547	0.825	0.795 ± 0.069 b	0.877	1.973	1.257 ± 0.255 b
wt-	15		1.117	1.379	1.254 ± 0.089 a	2.136	3.457	2.974 ± 0.327 a

^a^N:total number of T_4 _plants evaluated.

^b^Min: minimum value.

^c^Max: maximum value.

^d^wt+: wild plant inoculated with TMV or CMV.

^e^wt-: wild plant inoculated with buffer.

^f^Mean value followed by the same letter do not differ significantly at *p *< 0.05 level.

**Figure 5:**
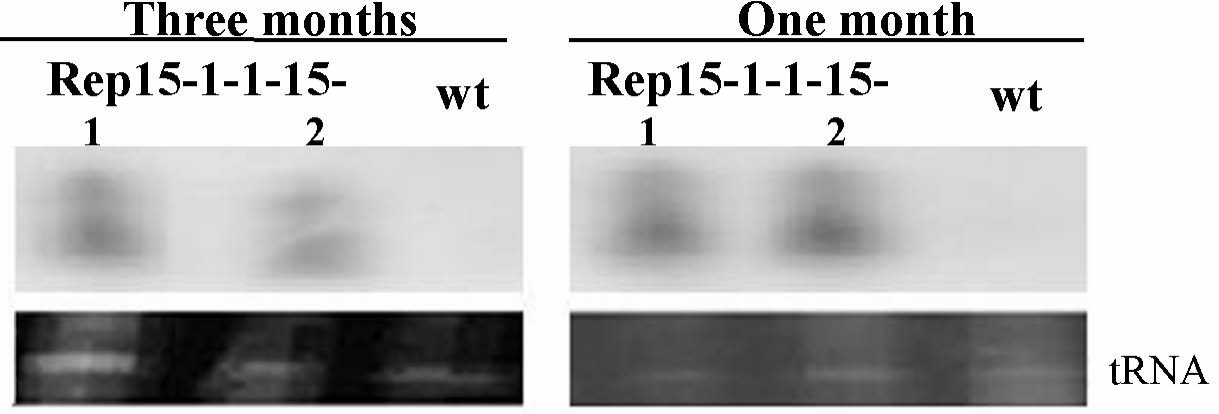
**Detection of CMV Rep specific siRNA at one or three months after virus inoculation in T**_**4 **_**transgenic lines Rep 15-1-1-15**. 1 and 2 represent two different T4 transgenic plants. wt represents wild plant. I, inoculated leaves; N, new growth leaves. The lower panel shows the loading level of each RNA sample by ethidium bromide staining.

Accumulation and composition of siRNAs at both one and three months after virus inoculation were compared, and results showed that there was little change of siRNAs at both one and three months (Figure 5). 21-24 nts siRNAs were at a high level at one month after virus inoculation, and the level of 21nts siRNA slight decrease but 24 nts siRNA level kept stable at three months after virus inoculation, which was supposed to play a role in systemic silencing and methylation of homologous DNA [10]. Thus, it seemed that the generation of transgene-specific siRNA could keep steady in the whole growth stage of T_4 _transgenic plants consistent to the resistance of T_4 _transgenic plants.

### RNA silencing-based virus resistance phenotypes were kept at low temperature

**Figure 6:**
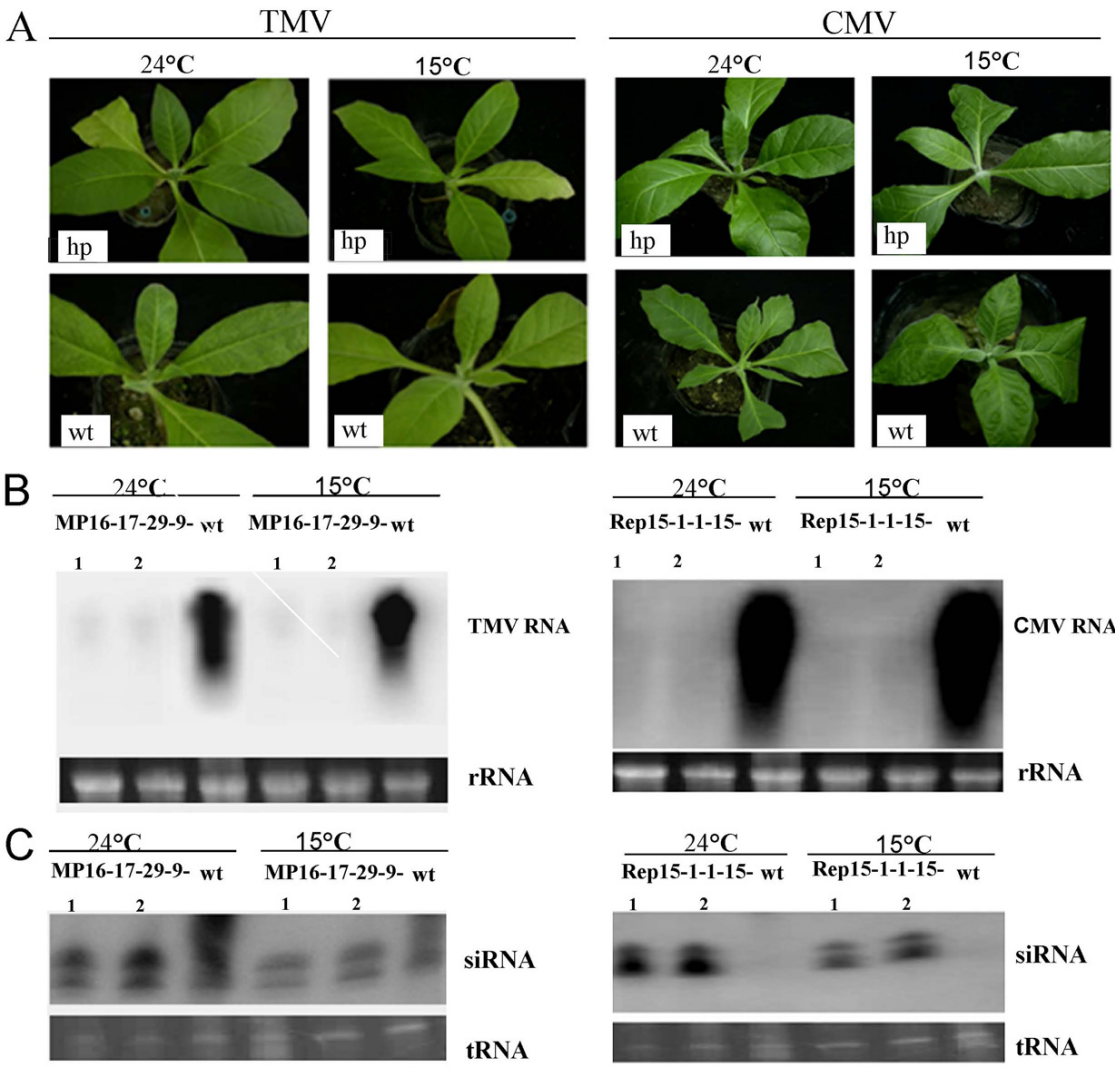
**Symptoms (A), viral RNA (B) and siRNA (C) accumulation levels of the transgenic plants expressing TMV hairpin MP RNA (left) or CMV hairpin Rep RNA (right) at 25 days after virus inoculation at 24℃or 15℃**. Transgenic plants (hp) and wild type (wt) plants were infected with TMV or CMV. Ribosomal RNA was applied as loading control.

To examine the effect of temperature on the virus resistance, the virus symptoms were observed and the virus RNA and siRNA of T_4 _progeny plants were detected at 24℃ and 15℃ at 25 days after TMV or CMV inoculation. Virus inoculation test showed that transgenic plants (MP16-17-29-9 or Rep15-1-1-15 lines) were immune to TMV or CMV at both 15℃ and 24℃ (Figure 6A). At 15℃, no any virus symptoms was developed and the virus RNA was low beyond a detected level (Figure 6B), siRNA was accumulated to a level as same as at 24℃ (Figure 6C), demonstrating that the transgene-mediated virus resistance was kept at low temperature.

### CMV infection did not break resistance to TMV in transgenic tobacco plants expressing TMV hairpin MP RNA

In order to know whether CMV can suppress the TMV silencing in TMV resistant transgenic plants, we carried out the following experiment. T_4 _progeny plants expressing TMV hairpin MP RNA were inoculated with TMV or CMV firstly, and then CMV or TMV at 25 days after TMV or CMV inoculation, or doubly inoculated with the two viruses at the same time. The TMV and CMV are subsequently detected by TAS-ELISA and Northern blot. Six weeks after inoculation, mosaic symptoms were observed on the upper leaves of the new emergent leaves of all inoculated transgenic plants, but not on the transgenic plants inoculated with TMV or buffer as controls (data not shown). TAS-ELISA results indicated that all the transgenic plants showing mosaic symptoms were infected by CMV (Table 4). No TMV was detected in inoculated transgenic tobacco plants, but was detected in untransformed tobacco plants. Northern blot analysis confirmed that TMV replicated to high level in all untransformed tobacco control plants, but to undetectable level in transgenic plants when co-inoculation with CMV and TMV (data not shown). The above results indicate that CMV could not break resistance to TMV in transgenic tobacco plants expressing TMV hairpin MP RNA.

**Table 4:** TAS-ELISA detection of T_4 _transgenic and wild type plants inoculated with TMV/CMV, CMV/TMV or TMV+CMV.

Challenge virus^a^	Plant lines	TMV		CMV	
		
		I^b^	N^b^	I	N
	MP16-17-29-7	0/10^c ^(0.054)^d^	0/10 (0.068)	10/10 (0.552)	10/10 (0.768)
TMV/CMV	MP16-17-29-9	0/10 (0.047)	0/10 (0.075)	10/10 (0.449)	10/10 (0.821)
	Wild plant	10/10 (0.778)	10/10 (0.829)	10/10 (0.578)	10/10 (0.813)

	MP16-17-29-7	0/10 (0.073)	0/10 (0.047)	10/10 (0.873)	10/10 (0.682)
CMV/TMV	MP16-17-29-9	0/10 (0.052)	0/10 (0.054)	10/10 (0.712)	10/10 (0.674)
	Wild plant	10/10 (0.852)	10/10 (0.852)	10/10 (0.748)	10/10 (0.652)

	MP16-17-29-7	0/10 (0.065)	0/10 (0.041)	10/10 (0.465)	10/10 (0.562)
TMV+CMV	MP16-17-29-9	0/10 (0.038)	0/10 (0.053)	10/10 (0.538)	10/10 (0.541)
	Wild plant	10/10 (0.754)	10/10 (0.882)	10/10 (0.564)	10/10 (0.518)

^a^TMV/CMV represents plants were inoculated TMV firstly and then inoculated with CMV on new emergent leaves at 25 days after TMV inoculation. CMV/TMV represents plants were inoculated CMV firstly, and then inoculated TMV on new emergent leaves at 25 days after CMV inoculation. TMV+CMV represents plants were inoculated with TMV and CMV at the same time.

^b^I represents inoculated leaves, N represents new emergent leaves

^c^Number of infected plants/number of inoculated plants.

^d^Number in brackets was average absorbance values of three independent ELISA assay.

## Discussion

Numerous examples of pathogen-derived resistance have been reported for a wide range of plant viruses. Transgenic plants expressing viral coat proteins have been successfully conferred the resistance to the corresponding viruses [1,11,12]. Expression of sequences corresponding to other viral genes have also become a successful tool for inducing pathogen-derived resistance, such as replicase gene [13-16], protease gene [17,18] and movement protein gene [19-21]. Transgenic pants expressing dsRNA by introduction of an inverted repeat sequence, spaced by an intron, into plants could reach 90% efficiency of gene silencing [22,23]. An effective method for obtaining resistant transgenic plants is therefore to induce RNA silencing by expressing virus-derived dsRNA in plants and this method has been successfully implemented for the generation of different plant lines resistant to many viruses [7,9,24-30]. We have demonstrated that transgenic tobacco plants expressed partial TMV movement gene or CMV replicase gene in the form of an intermolecular intron-hairpin RNA exhibited complete resistance to TMV or CMV infection. Due to the dsRNA nature, engineered specific RNA molecules were targeted for degradation, so only small steady-state amounts of the actual hairpin transcripts could be expected in the transgenic lines [28,31,32]. Our results also showed only very little transcript of the transgene could be detected after or before virus inoculation. Occurrence of siRNA is one of the most important characteristics of RNA silencing and can be a reliable molecular marker that is closely associated with viral resistance in transgenic plants expressing viral genes [31,33,34]. We also found siRNAs characteristic to RNA silencing were detected to accumulate in high levels in resistant transgenic plants whether virus was inoculated or not. These results indicated that TMV or CMV resistance observed in the resistant transgenic tobacco plants is attributed to RNA silencing.

Multiple complex patterns of transgene integration have been detected in many species such as tomato [28], cereal [7,35] and wood perennial tree (*Prunus domestica*) [36]. No general conclusions can be made as to whether a second copy of the transgene would increase the likelihood of virus resistance [31], so it is suggested no correlation between the copy number of insertions and types of RNA silencing [36,37]. We also found no correlation between the resistance and the copy number of the transgene.

Kalantidis K *et al*. [24] reported the concentration of siRNA reached a plateau at 30 days post-germination (one month) and then remained stable in the course of further development (two months). But Missiou *et al*. [31] reported that the accumulation and composition of transgene-specific siRNA was changed when plants were grown. Our results showed that there was little change of accumulation and composition of siRNAs at both one and three months after virus inoculation.

Plant-virus interactions are strongly modified by environmental factors, especially by temperature. High temperature is frequently associated with attenuated symptoms and with low virus content [38]. But rapid spread of virus disease and development of severe symptoms are frequently associated with low temperature [39]. Studied have shown that low temperature inhibited the accumulation of siRNAs in insect, plant and mammalian cells [10,40,41]. At low temperature, RNA silencing induced by virus or transgene was inhibited, which leads to enhancing virus susceptibility, to loss of silencing-mediated transgenic phenotypes and to dramatically reducing the level of siRNA, but the accumulation level of miRNA was not influenced by temperature [10]. So RNA silencing-based transgenic phenotypes were reported to be lost at low temperature (15°C). We found that RNA silencing-based transgenic phenotypes were not lost at low temperature (15°C). The virus siRNAs level was stable at both 24°C and 15°C and no obvious decrease of virus siRNAs accumulation was found at 15°C as compared with that at 24°C. Bonfim et al. [26] reported that the amount of siRNA at 25 °C showed a slight decrease as compared with that at 15 °C compared, but they did not test whether the resistance of transgenic bean plants with an intron-hairpin construction was influent. The differences of low temperature on RNA silencing-based transgenic phenotypes were unknown.

The PTGS pathway can be inhibited by suppressors encode by plant viruses [42,43]. The 2b protein of CMV suppresses PTGS by directly interfering with the activity of the mobile silencing signal [44,45]. Guerini and Murphy [46] reported that *Capsicum annum *cv. Avelar plants resisted systemic infection by the Florida isolate of Pepper mottle potyvirus (PepMoV-FL). However, co-infection of Avelar plants with CMV alleviated this restricted movement, allowing PepMoV-FL to invade young tissues systemically. Our results showed that the TMV-resistant transgenic tobacco plants were clearly not impacted by the suppressor, the 2b protein of CMV.

It's clear that regardless of the mechanistic details, the expression of viral dsRNA seems to be a highly efficient way to engineer virus-resistant plants, and the resistance induced by the hairpin RNA can be stably inherited through self-pollination for the fourth generations. Through this strategy, we can select for the most promising lines that are immune to viruses. Besides the high efficiency for generating transgenic plants resistant to a viral pathogen, the RNA-mediated resistance is good for environmental biosafety over the different protein mediated resistance as potential risks of heterologous encapsidation and recombination of virus are diminished.

## Conclusions

We expressed the partial TMV movement protein (MP) gene and the partial CMV replication protein (Rep) gene in the form of an intermolecular intron-hairpin RNA in transgenic tobacco. We analyzed the resistance of T_0 _to T_4 _transgenic plants. We found that T_4 _transgenic lines with single copy were completely resistant to the corresponding virus, and viral resistance of transgenic plants did not be affected by the low temperature (15℃). No significant correlation between the resistance and the copy number of the transgene was found. CMV infection could not break the resistance to TMV in the transgenic tobacco plants expressing TMV hairpin MP RNA.

## Methods

### Plant material and viruses

*Nicotiana tabacum *cv. Yunyan 87 was provided by Dr. Liu Yong (Yunnan Institute for Tobacco Science, China). TMV and CMV were isolated by the author's laboratory and maintained on *Nicotiana tabacum *cv. Xanthi nn in greenhouse.

### Construction of plant expression plasmids

Plant expression vector pBIN-TMVΔ*MP*(i/r), which contains inverted repeats of partial TMV *MP *gene (Δ*MP*) separated by the soybean intron was constructed previously [47]. For plant expression plasmid containing inverted repeats of CMV partial *Rep *gene (*ΔRep*) (Figure 1), specific primersΔ*Rep*-F (CGGTCGACGATAACTAAGTGGTGG, underline was *Sal*I site) and Δ*Rep*-R (CGATCGATCCAGACTTCTTGTATTTC, underline was *Cla*I site) designed according to the published CMV *Rep *gene (D00355) were used for PCR amplification using the plasmid pFny209 containing CMV *Rep *gene (kindly provided by professor Jialin Yu, China Agriculture University) and the amplified fragments were inserted into pUCm-T (Shanghai Sango, Shanghai, China) to produce recombinant plasmids pUCm-ΔRep(as) (antisense) and pUCm-Δ*Rep*(s) (sense), respectively. The plasmid pSK-In-ΔRep containing soybean intron and antisense Δ*Rep *fragment was obtained by digesting pUCm-Δ*Rep*(as) with *Pst*I and *Bam*HI and inserted into the vector pSK-In (kindly provided by professor Johansen, Danish Plant and Soil Graduate School) between the *Pst*I and *Bam*HI site. The plasmid pSK-In-*ΔRep *was digested by *Sal*I and *Bam*HI, and inserted into the *Sal*I and *Bam*HI site of the plant expression vector pBIN438 to produce recombinant expression vector pBIN-In-CMVΔ*Rep*. The sense Δ*Rep *fragment was obtained by digesting pUCm-Δ*Rep*(s) with *Sal*I, and then inserted into the *Sal*I site of pBIN-In-CMVΔ*Rep *to produce recombinant plant expression vector pBIN-CMVΔ*Rep*(i/r) (Figure 1), containing inverted repeats sequence of CMV Δ*Rep *separated by the soybean intron.

### Plant transformation, PCR and Southern blot analysis

The recombinant vector pBIN-TMVΔ*MP*(i/r) or pBIN-CMVΔ*Rep*(i/r) was transformed into *Agrobacterium tumerfaciens *EHA105, respectively, by the tri-parental mating method [48] and transgenic *Nicotiana tabacum *cv. Yunyan 87 plants were obtained using a leaf disc method as described [47]. Rooted plants were subsequently transferred to soil and grown to maturity in a greenhouse. Following self-fertilization of T_0_, T_1_, T_2_, T_3_, T_4 _progenies were tested for antibiotic sensitivity by rooting the seedlings on 50 mg/L of kanamycin. The presence and copy number of integrated intron-hairpin construction in selected tobacco transgenic lines were assessed by PCR and Southern blot. Tobacco genomic DNA was extracted from both the transgenic and non-transgenic leaf tissues (3 g) by the CTAB method [49], and analyzed for the presence of *MP *or *Rep *gene by PCR with primers TMV MP-F1 and TMV MP-R1 specific for TMV *MP *[47] and primers Δ*Rep*-F andΔ*Rep*-R specific for CMV *Rep*. Genomic DNA extracted from the PCR-positive plants (20-30 μg) was completely digested with *Dra*I or *Eco*RI or *Eco*RV, fractionated in 0.8% agarose gels and transferred onto Hybond N+ nylon membranes (Amersham Biosciences, Bucks, UK). DNA was cross-linked to the membrane using an UL-1000 ultraviolet crosslinker (UVP, Upland, CA, USA). Hybridization was conducted as described [50] using the [**α**-^32^P]dCTP-labelled TMV *MP *or CMV *Rep *gene as probe prepared by random primer procedure according to the Prime-a-Gene Labeling System (Promega, Madison, WI, USA).

### Virus resistance assays

Transgenic plants and wild plants were grown in greenhouse condition for 5 weeks before virus inoculation. Plants were mock-inoculated with phosphate buffer or inoculated with leaves sap extracts [diluted in 0.02 M phosphate buffer (pH 7.2)] from plants infected with TMV, CMV or both TMV and CMV (TMV+CMV). The inoculated plants were observed for virus symptoms after virus inoculation.

### TAS-ELISA

Leaf tissues (0.1 g) from new emergent leaves of each plant infected with TMV, CMV, TMV+CMV or inoculated with buffer were collected at 15, 25, 45 dpi. The virus concentration in the inoculated plants was detected by triple antibody sandwich enzyme-linked immunosorbent assay (TAS-ELISA) as described [51]. The absorbance values were measured in a Model 680 Microplate Reader (BIO-RAD, Hercules, CA, USA) at 405 nm.

### RNA isolation and analysis

Plants tissues were ground to a fine powder in liquid nitrogen and RNAs were extracted with TRIzol (Invitrogen, Grand Island, N.Y., USA), according to the manufacturer's instructions. The same RNA extract was separated to high- and low-molecular-mass RNAs using 30% PEG (molecular weight 8000, Sigma, Santa Clara, CA, USA) and 3 M NaCl as described [52]. The high-molecular-mass RNAs (20 μg) from transgenic plant tissues were separated on a 1% formaldehyde agarose gel and transferred to Hybond N+ nylon membranes (Amersham Biosciences) for Northern blot analysis. The low-molecular-mass RNAs (15 μg) were separated on a 15% sodium dodecyl sulfate (SDS) polyacrylamide gel with 7M urea and transferred to Hybond-N+ nylon membranes (Amersham Biosciences) by electrophoresis transfer at 400 mA for 45 min using a Bio-Rad semidry Trans-Blot apparatus. To verify equal amounts of siRNAs in each lane, gels also were stained with SYBR^® ^Gold nucleic acid gel stain (Invitrogen). Membranes were hybridized as described [50] with [**α**-^32^P]dCTP-labelled *MP *or *Rep *gene as probe prepared by random primer procedure according to the Prime-a-Gene Labeling System (Promega) overnight at 40℃ in 50% formamide buffer. 10-min three time post-hybridization washes were performed sequentially at 40℃ with 1× sodium chloride-sodium citrate buffer (SSC) supplemented with 0.1% SDS. Hybridization signals were detected by phosphorimaging using a Typhoon 9200 imager (GE Healthcare, Piscataway, NJ, USA).

## Competing interests

The authors declare that they have no competing interests.

## Authors' contributions

QH, YN, KZ, YL performed the experiments. QH, XZ analyzed the data and drafted the manuscript. XZ provided overall direction and conducted experimental design, data analysis and wrote the manuscript. All authors read and approved the final manuscript.
